# How does portfolio use affect self-regulated learning in clinical workplace learning: What works, for whom, and in what contexts?

**DOI:** 10.1007/s40037-022-00727-7

**Published:** 2022-09-22

**Authors:** Rozemarijn van der Gulden, Angelique Timmerman, Jean W. M. Muris, Bart P. A. Thoonen, Sylvia Heeneman, Nynke D. Scherpbier-de Haan

**Affiliations:** 1 Department of Primary and Community Care, Radboud University Medical Center, Nijmegen, The Netherlands; 2 Department of Family Medicine, Maastricht University, Maastricht, The Netherlands; 3 Department of Pathology, Maastricht University, Maastricht, The Netherlands; 4 Department of General Practice and Elderly Care Medicine, University Medical Centre Groningen, Groningen, The Netherlands

**Keywords:** Portfolio, Self-regulated learning, Realist review

## Abstract

**Introduction:**

Portfolio use to support self-regulated learning (SRL) during clinical workplace learning is widespread, but much is still unknown regarding its effectiveness. This review aimed to gain insight in the extent to which portfolio use supports SRL and under what circumstances.

**Methods:**

A realist review was conducted in two phases. First, stakeholder interviews and a scoping search were used to formulate a program theory that explains how portfolio use could support SRL. Second, an in-depth literature search was conducted. The included papers were coded to extract context–mechanism–outcome configurations (CMOs). These were synthesized to answer the research question.

**Results:**

Sixteen papers were included (four fulfilled all qualitative rigor criteria). Two primary portfolio mechanisms were established: documenting as a moment of contemplation (learners analyze experiences while writing portfolio reports) and documentation as a reminder of past events (previous portfolio reports aid recall). These mechanisms may explain the positive relationship between portfolio use and self-assessment, reflection, and feedback. However, other SRL outcomes were only supported to a limited extent: formulation of learning objectives and plans, and monitoring. The partial support of the program theory can be explained by interference of contextual factors (e.g., system of assessment) and portfolio-related mechanisms (e.g., mentoring).

**Discussion:**

Portfolio research is falling short both theoretically—in defining and conceptualizing SRL—and methodologically. Nevertheless, this review indicates that portfolio use has potential to support SRL. However, the working mechanisms of portfolio use are easily disrupted. These disruptions seem to relate to tensions between different portfolio purposes, which may undermine learners’ motivation.

**Supplementary Information:**

The online version of this article (10.1007/s40037-022-00727-7) contains supplementary material, which is available to authorized users.

## Introduction

The use of portfolios to support self-regulated learning (SRL) is common practice in medical education [1, 2]. Portfolios are a purposeful aggregation of (digital) items (e.g., evidence, reflections, feedback) that demonstrate learning, experience, or professional growth [3, 4]. SRL refers to “*the degree to which students are metacognitively, motivationally, and behaviorally active participants in their own learning process*” [5]. The literature indicates that higher levels of SRL are associated with better academic performance and lifelong learning [5, 6]. However, effective SRL is not self-evident, especially during clinical workplace learning (WPL), since it is difficult for learners to monitor their individual learning needs in the unpredictable and complex clinical setting [6, 7]. It is assumed that portfolios can mitigate this difficulty [4, 8].

Previous reviews have examined portfolio use for a variety of purposes, including competency development and assessment [1, 2, 4, 9]. With regard to SRL-related outcomes, the reviews are most informative about reflection. Although portfolio use was associated with an increased incidence of reflection, the quality of reflection did not necessarily improve with portfolio use [1]. This might be due to learners’ reluctance to disclose their introspections in a document accessible to faculty members who can influence their study prospects [2, 9]. Furthermore, some evidence suggests that portfolio use can support self-assessment and identification of learning needs [1, 2, 4, 9]. However, it was also shown that the use of a portfolio alone is not sufficient for these processes to occur, as several preconditions for successful portfolio use were mentioned, such as encouragement by a mentor [2, 4, 9] and clear portfolio goals and instructions [9]. Given the popularity of portfolios, it is important to gain insight in the extent to which and under what circumstances portfolio usage is effective for supporting SRL.

Therefore, we conducted a realist review to better understand when and how portfolio use supports SRL during clinical WPL. We used the following research question: How does portfolio use affect SRL during clinical WPL: What works, for whom, and in what contexts?

## Methods

A realist review is suitable to provide a rich and practice-oriented understanding of complex social interventions, such as portfolio use [10]. The aim of realist reviews is “*to unpack the mechanism of how complex programmes work (or why they fail) in particular contexts and settings*” [10]. To do so, the first step is to formulate a program theory, that can explain why the program under review is expected to work. Subsequently, literature is included to search for context–mechanism–outcome configurations (CMOs). In other words: what works for whom in which circumstances? Finally, a synthesis of these CMOs provides insight into the contexts and mechanisms that can explain different outcomes of the program. This approach fitted our aim to better understand when and how portfolio use supports SRL during clinical WPL.

### Review process

The review process consisted of two phases, which are described in more detail below (see Electronic Supplementary Material 1 [ESM 1] for a visualization of the review process). Two reviewers (AT, RG) performed the data collection and analysis. They discussed their approach and dilemmas that arose during the review process on a regular basis with the other authors. The standards of the RAMESES project were used to guide our decisions [11].

#### Phase 1

The goal was to formulate a program theory, which describes how portfolio use is expected to support SRL during clinical WPL.

##### Step 1: Stakeholder interviews

We conducted individual interviews to gather ideas and experiences from portfolio users. We included eight stakeholders from different institutes of the Dutch family practice specialty training, all of whom had previous experience with portfolio use and/or guidance. RG performed the interviews using a semi-structured interview guide. The interviews were audio recorded and later summarized. (See ESM 2 for information on the interviews.)

##### Step 2: Exploratory scoping search

Simultaneously with the stakeholder interviews, we performed a scoping search of PubMed and Web of Science in collaboration with a librarian to gather papers that explain how portfolio use supports SRL (July 2018; See ESM 3 for search strings). We selected search terms that included portfolios and SRL or self-directed learning (SDL) during WPL. Although there are intrinsic differences between SRL and SDL, we included both, given the interchangeable use of these terms in the literature [12]. The search resulted in 53 references; 45 references remained after removing duplicates. We considered 14 papers useful to formulate a program theory, as these papers theorized about how portfolio use can support SRL [13–26].

##### Step 3: Formulating the program theory

**Fig. 1:**
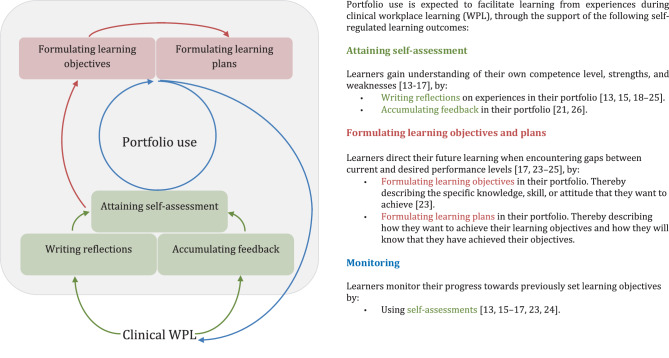
The program theory that describes how portfolio use is expected to support SRL during clinical WPL

To establish a first version of the program theory, RG and AT examined the interview summaries and included papers to extract ideas and theories that explain how portfolio use supports SRL. Subsequently, the theory was clarified and adapted in discussion with the other authors. During these discussions we recognized that interviewees and papers often (implicitly) assumed a relationship between portfolio use and the completion of learning cycles. The stages of the learning cycles explained by interviewees and in the included papers are similar to the experiential learning cycle described in Kolb’s theoretical framework [27]. Therefore, we utilized this experiential learning cycle as middle range theory. This resulted in a final version of the program theory provided in Fig. 1.

#### Phase 2

The goal was to provide an overview of the current research regarding portfolio use for the support of SRL during clinical WPL.

##### Step 4: In-depth literature search

The in-depth literature search (February 2019) was supported by the librarian who had also assisted during the scoping search. The original search strings were revised to also retrieve references regarding the SRL outcomes that are part of the program theory, e.g., self-assessment and learning cycle (ESM 4). We searched: Pubmed, CINAHL, ERIC, PsycInfo, Embase, and Web of Science.

##### Step 5a: Title and abstract screening

RG and AT screened title and abstract of the references for inclusion, which required that references needed to concern primary research into portfolio use for the support of SRL/SDL (or related outcomes) during clinical WPL. The first 300 references were assessed and discussed by both reviewers to establish the approach and definitive list of inclusion criteria (ESM 5). The remaining 1744 references were assessed by one reviewer. However, references that raised any doubts were discussed between the two reviewers.

##### Step 5b: Full-text review

The remaining papers were read and assessed by one of the two reviewers, and discussed if there were any doubts regarding inclusion.

##### Step 6: Assessing rigor

We evaluated the rigor of the included papers, as is prescribed by the realist tradition, to provide information on the credibility and trustworthiness of the papers [28]. We discussed information on quality assessment criteria and procedures used in other realist reviews [28–30], to compose the following criteria for the rigor evaluation:

1. There is a clear statement of, and rationale for, the research question/aims.
2. Design and study methods are appropriate to answer the research question.
3. Study findings and conclusions are supported by the data.

RG paired with the other authors to decide whether the included papers met these criteria. The criteria were evaluated individually first, and later discussed between the pairs to reach consensus.

##### Step 7: Extracting CMOs

The research question guided the formulation of applied definitions for context, mechanism, and outcome:

- Context: The external factors that affect portfolio use for the support of SRL. These factors could still exist without the portfolio present.
- Mechanism: The processes set in motion by portfolio use that influence the degree and/or level of SRL. These processes would not exist without the portfolio present.
- Outcome: SRL that is generated by portfolio use.

The reviewers individually coded the included papers for phrases that described contexts, mechanisms, or outcomes as defined above. We only included phrases directly linked to the study that was performed by the authors of the paper, as the focus was on primary data. Subsequently, the reviewers discussed their coding to reach consensus about the CMOs present. It was decided to also include incomplete CMOs, as these can help to clarify how portfolio use works for the support of SRL. Multiple iterations of coding and discussion were performed to formulate the final CMOs. Coding was supported by NVIVO (https://www.qsrinternational.com/nvivo) and Atlas.ti (https://atlasti.com/).

##### Step 8: Synthesizing CMOs

To synthesize the CMOs, we visualized each CMO by putting arrows between its context, mechanism and outcome with the use of mindmapping software (http://www.mindomo.com). This enabled us to easily (re)order different contexts, mechanisms and/or outcomes inductively, without losing the connection between individual CMOs. In that way, we could identify overarching contextual factors by grouping similar contexts together. Likewise, similar mechanisms were abstracted into overarching portfolio (related) mechanisms. We deductively organized the outcomes of the CMOs according to the SRL outcomes of the program theory: self-assessment, reflection, feedback, learning objectives and plans, and monitoring. Finally, we used the identified contextual factors, portfolio (related) mechanisms, and SRL outcomes to compose a model that illustrates how portfolio use supports SRL during clinical WPL.

## Results

### Study characteristics

We included 16 papers (see ESM 6 for a flowchart of the extraction process) [31–46]. These papers originated from eleven countries across Africa (2), Asia (3), Europe (8) and North America (3). Studies were conducted in undergraduate medical settings (5) and postgraduate ones (11). Most papers describe an evaluation of portfolio implementation (14). Although quantitative, qualitative and mixed methods designs are present, the questionnaire was the most popular method of data collection (10). Only four papers fulfilled the three rigor criteria (see ESM 7 for a more elaborate description of the included papers).

### Overview of identified CMOs

An overview of the identified CMOs is provided in Tab. 1. The different CMOs are organized according to the SRL outcome they were assigned to.

**Table 1:** The context–mechanism–outcome configurations identified during the extraction process of the 16 included papers focusing on portfolio use in clinical workplace learning (WPL), ordered according to SRL outcomes

Context^a^	Mechanism^b^	Outcome^c^
It was difficult to ensure protected time within the clinical hospital setting (WPL):	Learners thought that time spent on the portfolio reduced the time available to spend on patients (AP)	Learners doubted the educational benefit of the portfolio [42]
– Learners did not have a personal work and/or storage space within the hospital (WPL) – The busy, frenetic pace of the clinical setting (WPL) – Summative assessment; more specifically, requirements regarding the (number of) portfolio reports (SA)	Learners struggled to collect the required portfolio forms, because they usually did not have access to their portfolio at the workplace. But also because they were reluctant to add to the workload of colleagues by asking them to observe and provide feedback on routine procedures (M). Consequently, the portfolio requirements induced stress, anxiety, and other negative feelings (F)	Learners doubted the educational benefit of the portfolio [39]
*Context*	*Mechanism*	*Outcome* **Self-assessment (+)**
The busy, frenetic pace of the clinical setting, which can result in the training year passing by without any concrete developments (WPL)	The portfolio provided a structure to document information during busy workdays (DC). Subsequently, this documentation reminded learners of what happened before and thus provided an opportunity to look back (DR)	Learners reviewed their weak and strong points [44]
	Learners documented frank and open portfolio reports about their deficiencies and how they had tried to remedy them (DC)	Learners were aware of their feelings, attitudes and concerns [37]
	Distilling clinical experiences into portfolio reports helped to analyze these experiences (DC)	Learners engaged in constructive self-criticism, thereby clarifying thoughts and feelings and identifying proficiencies and deficiencies in performance [35]
	The portfolio facilitated that all information was stored in one place (DR)	Learners identified gaps in learning [32]
*Context*	*Mechanism*	*Outcome* **Self-assessment (−)**
– Learners did not have a personal work and/or storage space within the hospital (WPL) – The busy, frenetic pace of the clinical setting (WPL) – Summative assessment; more specifically, requirements regarding the (number of) portfolio reports (SA)	Learners struggled to collect the required portfolio forms, because they usually did not have access to their portfolio at the workplace. But also because they were reluctant to add to the workload of colleagues by asking them to observe and provide feedback on routine procedures (M). Consequently, the portfolio requirements induced stress, anxiety, and other negative feelings (F)	Learners did not experience the portfolio as help in the identification of strengths and weaknesses in one’s own performance [39]
*Context*	*Mechanism*	*Outcome* **Reflection (+)**
– Time-pressure (WPL) – Upcoming job interviews (WPL/A)	Learners documented short, superficial, selective and/or strategic portfolio reports (DC), because of time constraints, the idea that the portfolio was a record of achievement (AP), and privacy concerns (CP). Nevertheless, these short notes helped in remembering the events that had taken place (DR)	Although documented reflections were superficial, learners could engage in deep reflection at a later moment when they were reminded of the events that had taken place [33]
The busy, frenetic pace of the clinical setting (WPL)	Brief dedicated time to write in the portfolio needed to be secured (CP)	Reflections could be captured as they happened [41]
The busy, frenetic pace of the clinical setting (WPL)	Learners experienced limited time for reflection. Yet portfolio writing was considered to take up no additional time, as it was possible to do this in between the regular tasks and responsibilities (AP)	Learners engaged in reflection [44]
The busy, frenetic pace of the clinical setting made it difficult to synthesize learning experiences (WPL)	However, documenting in the portfolio provided an intentional deliberate moment to pause and think about what had happened during the day (DC)	Learners engaged in reflection [43]
	Learners documented frank and open portfolio reports about their deficiencies and how they had tried to remedy them (DC)	Learners engaged in reflection [37]
	Distilling clinical experiences into portfolio reports helped to analyze these experiences (DC)	Learners engaged in reflection [35, 42]
	The portfolio helped to capture what was happening during education (DC)	Learners engaged in reflection [45]
	The portfolio mimicked authentic professional situations since real medical cases were used to fill the portfolio. This reduced the gap between theory and practice (DR/PC)	Learners engaged in reflection [31]
	The portfolio facilitated that all information was stored in one place (DR)	Learners engaged in reflection [32]
	Supervisors emphasized the importance of reflection (M)	Learners engaged in reflection [33]
*Context*	*Mechanism*	*Outcome* **Reflection (−)**
– Low resource country with scarce human resources (GC) – Learners and supervisors were not aware of nor experienced with reflective thinking and writing. (ES)	Learners thought that they should not reveal any weaknesses, deficiencies, or mistakes in their portfolio and should only include evidence of competency (AP)	Although reflection was happening, it was almost unconscious, and very seldom documented [40]
*Context*	*Mechanism*	*Outcome* **Feedback (+)**
Eastern face-saving culture (GC)	Learners feared that (negative) feedback would result in uncomfortable situations with teachers (I/F). However, the digital portfolio format provided learners the opportunity to read feedback when alone, which diminished fear (PC/F)	Learners were able to learn from the feedback that was provided [36]
	Sensitive or otherwise neglected topics were discussed more easily as they were part of the portfolio and needed to be discussed. This broadened the focus of discussions between learners and supervisors and/or stimulated the relationship between them (M)	Honest and constructive feedback between learner and supervisor was facilitated [44, 45]
	Supervisors were able to provide valuable feedback when learners documented complete portfolio reports (M). Feedback was considered valuable by learners in case it concerned specific clinical cases, as this feedback promoted further thought and actions (AP). Moreover, learners enjoyed it when supervisors paid enough attention to provide them with individualized feedback, which promoted a positive self-image of learners (F)	Learners actively sought and kept feedback. Provided feedback was used retrospectively (i.e., reading documented feedback to seek solutions after a problem is encountered) and prospectively (i.e., changing practice after reading feedback) [36]
	Supervisors were able to provide valuable feedback when learners documented complete portfolio reports (M)	Feedback promoted further research and thought into a topic [46]
*Context*	*Mechanism*	*Outcome* **Feedback (−)**
The number of interesting cases available in the clinical setting varied (WPL)	When there was a lack of interesting cases, learners wrote brief reports in their portfolio. These brief reports were followed by brief feedback from supervisors (M)	Learners perceived portfolio feedback to have little utility for personal development and, as a result, their feedback-seeking motivation declined [36]
Unsatisfying technological infrastructure at the hospital (WPL)	Learners were forced to complete portfolio entries at home after work. Due to the time delay learners experienced difficulties in writing optimal submissions. These submissions received suboptimal feedback in return (M)	Learners perceived portfolio feedback to have little utility for personal development and, as a result, their feedback-seeking motivation declined [36]
The heavy clinical workload of supervisors (WPL)	Supervisors often did not react (in due time), as there was no reminder function in the portfolio. As a consequence, trainees (repeatedly) checked their portfolio without finding any feedback, which resulted in frustration (PC/F)	Learners perceived portfolio feedback to have little utility for personal development and, as a result, their feedback-seeking motivation declined [36]
Workplace learning (WPL)	Feedback was already provided during bedside teaching (M)	Learners perceived portfolio feedback to have little utility for personal development and, as a result, their feedback-seeking motivation declined [36]
Summative assessment; more specifically, requirements regarding the (number of) portfolio reports (SA)	The mandatory number of submissions resulted in a high frequency of feedback. Therefore, single feedback moments had low news value (M)	Learners perceived portfolio feedback to have little utility for personal development and, as a result, their feedback-seeking motivation declined [36]
Learners found it difficult to perform a learning needs assessment individually (ES)	Learners experienced a lack of guidance and clear instructions on how to complete the portfolio (M/PC). As a consequence, trainees felt uncertain about what they should write in their portfolios (F) and were inclined to make inauthentic submissions. This type of submission evoked superficial or generic feedback by teachers (M)	Learners perceived portfolio feedback to have little utility for personal development and, as a result, their feedback-seeking motivation declined [36]
	Due to the number of trainees rotating between the departments and the genericity of provided feedback, learners distrusted supervisors’ ability to remember individual learners and their performance well enough to provide individualized feedback (I/M)	Learners perceived portfolio feedback to have little utility for personal development and, as a result, their feedback-seeking motivation declined [36]
	Delayed feedback was experienced as less meaningful. Since learners believed that supervisors were not able to remember the past experience well enough to provide accurate feedback (I/M)	Feedback-seeking motivation declined [36]
*Context*	*Mechanism*	*Outcome* **Learning objectives and plans (+)**
The busy, frenetic pace of the clinical setting made it difficult to synthesize learning experiences (WPL)	However, documenting in the portfolio provided an intentional deliberate moment to pause and think about what had happened during the day (DC)	Learners thought how lessons learned could be applied in the future [43]
	Distilling clinical experiences into portfolio reports helped to analyze these experiences (DC)	Learners directed future progression, as they charted their own course of professional development [35]
	The portfolio facilitated that all information was stored in one place (DR)	The portfolio helped to set goals for the future [32]
*Context*	*Mechanism*	*Outcome* **Learning objectives and plans (−)**
– Learners did not have a personal work and/or storage space within the hospital (WPL) – The busy, frenetic pace of the clinical setting (WPL). – Summative assessment; more specifically, requirements regarding the (number of) portfolio reports (SA)	Learners struggled to collect the required portfolio forms, because they usually did not have access to their portfolio at the workplace. But also because they were reluctant to add to the workload of colleagues by asking them to observe and provide feedback on routine procedures (M). Consequently, the portfolio requirements induced stress, anxiety, and other negative feelings (F)	Learners did not experience the portfolio as help in the achievement of learning objectives [39]
– Low resource country with scarce human resources (GC). – Learners and supervisors were not aware of and experienced with reflective thinking and writing (ES)	Learners thought that they should not reveal any weaknesses, deficiencies, or mistakes in their portfolio and should only include evidence of competency (AP)	Learners were reluctant to document learning needs [40]
*Context*	*Mechanism*	*Outcome* **Monitoring (+)**
The busy, frenetic pace of the clinical setting, which can result in the training year passing by without any concrete developments (WPL)	The portfolio provided a structure to document information during busy workdays (DC). Subsequently, this documentation reminded learners of what had happened before and thus provided an opportunity to look back (DR)	Learners looked back and mapped their development [44]
	Existing documentation of (learning) events provided the opportunity to reconsider past events (DR)	Learners gauged their own progress, as returning to previous reports helped to see what they had learned and how they had progressed [33]

^a^ The contextual factors are indicated as follows: characteristics of workplace learning (WPL), system of assessment (SA), geographical/cultural characteristics (GC), prior experience with SRL constructs (ES)

^b^ The portfolio (‑related) mechanisms are indicated as follows: documenting as a moment of contemplation (DC), documentation as a reminder of past events (DR), conditions of portfolio use (CP), mentoring (M), assumptions about portfolio use (AP), feelings about portfolio use (F)

^c^ The CMOs are ordered according to the SRL outcome they were assigned to during synthesis: self-assessment, reflection, feedback, learning objectives and plans, and monitoring. For each SRL outcome the positive outcomes are provided first (+) and the negative outcomes second (−)

### A model of how portfolio use works for the support of SRL during WPL

**Fig. 2:**
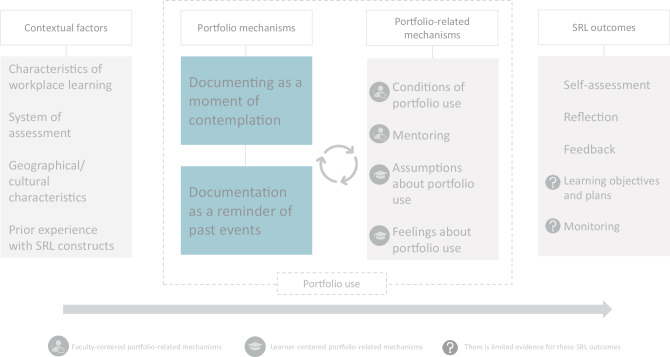
The relationship between contextual factors, portfolio (‑related) mechanisms and SRL outcomes

The relationship between contextual factors, portfolio (‑related) mechanisms and SRL outcomes is visualized in Fig. 2. Below we explain the components of the model.

#### Contextual factors

The contexts of the CMOs were abstracted into four contextual factors. First, we identified a relationship between portfolio use and *characteristics of workplace learning *[33, 36, 39, 41–44]. Multiple papers referred to aspects of WPL that can complicate learning, such as limited access to computers, time constraints resulting from a high workload and the frenetic pace of the clinical setting. Some papers explained that portfolio requirements added to these pressures [36, 39]. In contrast, other papers described that portfolio use resolved issues related to WPL by creating moments of contemplation, i.e., learners that were able to use their portfolio during busy workdays were provided with an opportunity to pause and think about what had happened [36, 39, 43, 44].

The second contextual factor concerns the *system of assessment* in place [36, 39]. Two studies showed that formal requirements concerning the amount and/or quality of portfolio reports in the context of summative assessment were related to stress and anxiety of learners [36, 39]. These assessment requirements might interfere with the support of portfolio use for SRL, as learners in one of these studies did not experience any educational benefits of the portfolio [39].

Two papers mentioned *geographical/cultural characteristics* in relation to portfolio use [36, 40]. The face-saving culture present in Taiwan was thought to influence feedback seeking of learners with the portfolio, due to a fear of negative feedback [36]. In addition, Jenkins et al. describe how the exceptionally high service demands in South Africa limited time available for portfolio use [40]. In the same two studies the final contextual factor surfaced: *prior experience with SRL constructs *[36, 40]. It was described that limited experience with reflection on the part of learners and supervisors resulted in low awareness and documentation of reflection in the portfolio [40]. Furthermore, the learners in the study of Fu et al. found it difficult to individually perform a learning needs assessment, which resulted in inauthentic portfolio reports [36].

#### Portfolio (-related) mechanisms

The mechanisms of the CMOs were distilled into:

- Portfolio mechanisms: primary mechanisms inherent to portfolio use that seem to affect SRL directly.
- Portfolio-related mechanisms: mechanisms related to portfolio use that seem to affect the primary portfolio mechanisms and thereby also SRL.

The first portfolio mechanism affecting SRL concerns *documenting as a moment of contemplation* [33, 35, 37, 42–45]. Documenting was reported to help learners analyze their experiences; writing a portfolio report helped learners to capture the essence of their experiences [35, 42, 43, 45]. The second portfolio mechanism is *documentation as a reminder of past events* [32, 33, 44]. Previously documented information helped learners to remember what happened before, which provided an opportunity to mentally return to these events [33, 44].

The first two portfolio-related mechanisms are controlled by training institutes and their faculty. First, some papers mentioned conditions of portfolio use (e.g. a digital format, the provided instructions, or privacy matters) that affected portfolio use and thus the potential for documenting and use of documentation [31, 33, 36, 41, 43, 44, 46]. The second portfolio-related mechanism controllable by training institutes and/or faculty concerns *mentoring* during portfolio use [33, 36, 39, 44–46]. Some papers describe how portfolio use supported mentoring and, in this way, SRL: feedback was exchanged more easily, since sensitive or otherwise neglected topics were included in the portfolio and therefore discussed [44, 45]. However, others described that supervisors could only provide valuable feedback when learners provided suitable portfolio reports [36, 46]. Also WPL (contextual factor) can interfere with the possibility of exchanging feedback through the portfolio: learners were hesitant to ask busy supervisors for portfolio contributions, as they did not want to add to the supervisor’s workload [39].

The other portfolio-related mechanisms concern different aspects of learners’ reactions to portfolio use. Multiple papers described learners’ *assumptions about portfolio use* [33, 36, 40, 42, 44]. Two papers showed that positive assumptions about the potential of the portfolio for reflection and feedback were related to the occurrence of these SRL outcomes [36, 44]. Likewise, Kjaer et al. identified negative assumptions about portfolio use during clinical care that related to doubts about the portfolio’s educational benefit [42]. Two other papers reported that learners only considered the portfolio suitable to provide evidence of competence and not for (extensive) reflection [33, 40].

Furthermore, two papers referred to learners’ *feelings about portfolio use* related to SRL [36, 39]. There were learners that experienced stress and anxiety in reaction to the system of assessment, which potentially limited SRL [36, 39]. In contrast, Fu et al. also mention positive effects of portfolio use on feelings. They mentioned, for example, how learners experienced a positive self-image when supervisors took the effort to provide them individualized feedback [36].

#### SRL outcomes

We assigned the outcomes of the CMOs to the SRL outcomes that are part of the program theory. The outcomes related to *self-assessment* were mostly encouraging [32, 35, 37, 39, 44]. Learners identified proficiencies and deficiencies in performance [32, 35, 44] and/or were aware of their own thoughts and feelings [35, 37] with help of their portfolio. One study described that the system of assessment (contextual factor) interfered with this process, as learners mainly experienced stress and anxiety in concern to portfolio assessment and did not experience any benefit for the identification of individual strengths and weaknesses [39].

A majority of the papers mentioned a relationship between portfolio use and learner *reflection* [31–33, 35, 37, 40–45]. While most papers stated that portfolio use promoted reflection, they often did not explain this relationship further [31, 32, 35, 37, 42, 45]. Brown et al. was an exception explaining that short portfolio reports that were jotted down in between tasks of busy workdays were accompanied by superficial reflection. However, deep reflection could be instigated at a later (quieter) moment, when the portfolio report reminded the learner of the event that took place [33].

Different activities concerning *feedback* were discussed as an outcome of portfolio use [36, 44–46]. Two papers described that honest and constructive feedback between learner and supervisor was facilitated by portfolio use, as sensitive or otherwise neglected issues were discussed more easily now that they were part of the portfolio process [44, 45]. Moreover, the interaction between provided feedback and feedback-seeking of learners was described by Fu et al., who showed that the perceived utility of feedback was positively related to feedback-seeking motivation. Learners perceived individualized feedback to have a high utility, whereas more general feedback was considered less useful [36].

The role of portfolio use was less convincing for *learning objectives and plans* [32, 35, 39, 40, 43]. We found some positive outcomes, e.g., thinking about the application of lessons learned in the future [32, 35, 43]. However, other papers reported difficulties related to portfolio use and learning objectives. Jenkins et al. found that learners were reluctant to document learning needs, as they did not want to show weaknesses [40]. In addition, learners described by Hrisos et al. did not experience the portfolio as helping with the achievement of learning objectives, as they struggled to collect the required portfolio reports [39].

Lastly, two papers provided results concerning *monitoring* [33, 44]. These papers reported a positive outcome related to portfolio use: learners in these studies were able to assess their progress, as they were able to look back on what they had learned because portfolio documentation acted as reminder of past events [33, 44].

## Discussion

We conducted a realist review to better understand when and how portfolio use supports SRL during clinical WPL. Based on the CMOs of 16 included papers, we developed a model to describe how contextual factors and portfolio (‑related) mechanisms can influence SRL outcomes. In contrast to the program theory, our realist review found limited evidence for the assumption that portfolio use supports the formulation of learning objectives and plans, and monitoring. Our findings do describe a relationship between portfolio use and the other SRL outcomes of the program theory: self-assessment, reflection, and feedback. However, these SRL outcomes were found in isolation, contrary to the program theory that describes a learning cycle in which the different SRL outcomes flow into each other.

The limited support for the program theory can be explained by characteristics of the included papers: learning objectives and monitoring were included less as outcome of interest than the other SRL outcomes, and the study designs were often not suitable to explore interactional relationships between SRL outcomes. However, our findings also indicate that the contextual factors in interaction with the portfolio-related mechanisms can easily disrupt the primary portfolio mechanisms—documenting as a moment of contemplation and documentation as a reminder of past events—and with that SRL.

The most prominent contextual factors that emerged from the CMOs were WPL in interaction with the assessment program: learners in a busy clinical setting can easily get overwhelmed by time constraints and/or the wish to perform well. But the portfolio-related mechanisms indicate that the pressures of WPL can be avoided by ensuring sufficient time and opportunities for learners to use the portfolio during workdays. In contrast, our results did not highlight any solutions for the tensions that result from (summative) portfolio assessment. This aligns with an ongoing debate about the desirability of multipurpose portfolios: should we use the portfolio for both assessment and to support SRL [2, 9]?

It is likely that tensions between these two potentially contrasting portfolio purposes undermine learner motivation and thereby limit SRL. While motivation is an important construct in many SRL theories [47], other motivation theories might help explain this relationship, such as self-determination theory (SDT) [48]. The foundation of SDT consists of a series of principles about human nature, that explain a continuum from amotivation to intrinsic motivation. SDT states that three innate needs must be fulfilled for intrinsic motivation to endure: the need for autonomy, the need for competence, and the need for relatedness with others. It is conceivable that portfolio assessment can frustrate the need for autonomy (freedom of choice is limited by the system of assessment) and for competence (during assessment one might be confronted with [fear of] incompetence), and thereby limits intrinsic motivation. Since intrinsic motivation is described by concepts such as autonomy and self-regulation and is associated with better learning [48], it is likely that a decline in intrinsic motivation is in essence a decline in SRL.

Goal orientation theory can also explain a detrimental effect of portfolio assessment on the support of portfolio use for SRL [49]. Although there are multiple versions of this theory, in general two classes of goal orientation are distinguished: a learning/mastery orientation (aims to increase competence) and a performance orientation (aims to gain favorable judgments of competence or avoid negative judgments of competence). It is conceivable that portfolio assessment can provoke a performance orientation in learners. This might limit SRL, as studies have shown that a performance orientation is negatively related to goal setting, feedback processes, and metacognition [49]. However, there is a SRL construct that seems to moderate this relationship: self-efficacy (the belief that one can complete a task successfully). For those with a high level of self-efficacy the initial approach of a task did not differ, regardless of goal orientation [49]. Consequently, self-efficacy might be a construct of interest within portfolio research, because of its connection with motivation.

To attain knowledge about the importance of motivation and self-efficacy for portfolio use, it is important that researchers delve more deeply into SRL. During our analysis, we noticed that most papers failed to define and/or operationalize the SRL outcomes included in their studies [31–34, 36, 38, 39, 41–46]. This is troublesome, as SRL and its related constructs are complex and encompass a variety of ideas and practices [50]. As long as we fail to explicate the SRL outcomes that we include in portfolio research, it will be impossible to attain a common understanding of what portfolio use can (not) achieve for the support of SRL.

Given the different gaps in the scientific knowledge base, it is still too early to consider practical implications. However, our results indicate that allotted time and opportunity for portfolio use during clinical WPL are important. Further research, in which (motivational) theory is adequately incorporated, is necessary to formulate clear recommendations with regard to portfolio use for the support of SRL.

### Implications for future research

First, it is important that future research provides adequate definitions and operationalizations of SRL. Second, research should move beyond the evaluation of portfolio implementations and empirically study the relationship between portfolio use and SRL. Specifically, in-depth qualitative approaches (e.g., observations or focus groups) would be interesting, as insight into individual experiences can help to explore the role of motivation and self-efficacy as potential drivers for portfolio use in relation to SRL outcomes.

### Study limitations

We conducted a realist review to provide a rich and contextualized overview of portfolio use for the support of SRL. However, to formulate CMOs a reductionist approach was sometimes needed, as we were unable to include all information present in the papers. Consequently, the presence of interactional relationships and/or simultaneously occurring processes was sometimes disregarded, and thus causality within individual CMOs can become overrated. Furthermore, as our literature search was conducted in 2019 it is possible that more recent publications could have provided additional and/or different insights than the ones provided in our model. Lastly, we call attention to the methodological weaknesses of the included papers. This was unsurprising as previous reviews have reported a similar lack of robustness in this domain [2, 9]. Consequently, we stress the tentative nature of our findings and the developed model.

## Supplementary Information

Electronic supplement 1 A visualization of the design of the realist review regarding portfolio use to support self-regulated learning in clinical workplace learning

Electronic supplement 2 Additional information concerning the stakeholder interviews

Electronic supplement 3 Search strings exploratory scoping search

Electronic supplement 4 Search strings in-depth literature search

Electronic supplement 5 Inclusion criteria in-depth literature search

Electronic supplement 6 Flowchart of the in-depth literature search and screening proces

Electronic supplement 7 Characteristics and qualitative rigor evaluation of the 16 included papers
