# Supplementary material for: How does portfolio use affect self-regulated learning in clinical workplace learning: What works, for whom, and in what contexts?
Source: Perspect Med Educ. 2022 Sep 22;11(5):247–57. doi: 10.1007/s40037-022-00727-7 (PMC9582105; doi:10.1007/s40037-022-00727-7)
Supplement: Supplementary file 1 — Electronic supplement 1 A visualization of the design of the realist review regarding portfolio use to support self-regulated learning in clinical workplace learning [file 40037_2022_727_MOESM1_ESM.docx]

**Electronic supplement 1**A visualization of the design of the realist review regarding portfolio use to support self-regulated learning in clinical workplace learning.

P
h
a
s
e

1

**Step 2**: Exploratory scoping search (July 2018)

**Step 1**: Stakeholder interviews (July 2018)

**Step 3:** Formulating the program theory (January 2019)

P
h
a
s
e

2

**Step 4:** In-depth literature search
(February 2019)

**Step 5a:** Title and abstract screening (November 2019) January 2019) (July018 – January 2019)

**Step 5b:** Full-text review (March 2020)

**Step 7:** Extracting CMOs (November 2020)

**Step 6:** Assessing rigor (August 2020)

**Step 8:** Synthesizing CMOs (July 2021)
