# Supplementary material for: How does portfolio use affect self-regulated learning in clinical workplace learning: What works, for whom, and in what contexts?
Source: Perspect Med Educ. 2022 Sep 22;11(5):247–57. doi: 10.1007/s40037-022-00727-7 (PMC9582105; doi:10.1007/s40037-022-00727-7)
Supplement: Supplementary file 2 — Electronic supplement 2 Additional information concerning the stakeholder interviews [file 40037_2022_727_MOESM2_ESM.docx]

**Electronic supplement 2**Additional information concerning the stakeholder interviews

Participants

|  | **Function** | **Institute** |
| --- | --- | --- |
| 1 | Faculty member (Behavioral scientist) | Radboudumc |
| 2 | Local Assessment coordinator | Radboudumc |
| 3 | Head of GP training institute | Leiden university medical center |
| 4 | Faculty member (General Practitioner) | Radboudumc |
| 5 | National Assessment coordinator | Network of 8 GP training institutes |
| 6 | Clinical supervisor | Radboudumc |
| 7 | Trainee (Year 3) | Radboudumc |
| 8 | Trainee (Year 2) | Radboudumc |

Interview guide

| Introduction:   - Provide short explanation regarding the purpose PhD project - State duration of the interview (+/- 45 minutes) - Ask for consent concerning the audio recording (start recording) - State purpose of the realist review - Provide definition of self-regulated learning: “*the degree to which students are metacognitively, motivationally, and behaviorally active participants in their own learning process”* - Provide explanation procedure (We will start with some broad questions about portfolio use. Thereafter, more focused questions will follow. There are no right or wrong answers, we are only interested in your experiences and perspectives.) - Ask if there are any questions or comments before we start? | | |
| --- | --- | --- |
| 1. | How do you view and experience the support of portfolio use for self-regulated learning? | |
| 2. | Which pros and cons do you notice concerning the use of portfolios for the support of self-regulated learning? | |
| 3. | Are there any requirements that need to be fulfilled in order to (effectively) use a portfolio for the support of self-regulated learning? | |
| After these more general questions I am curious to hear more about your personal experiences with (guidance of) portfolio use. | | |
| 4. | | To what extent do you understand the purpose and use of the portfolio? |
| 5. | | To what extent do trainees accept the portfolio? |
| 6. | | How do you experience the user-friendliness of the portfolio? |
| 7. | | To what extent can faculty impact portfolio use of trainees and their supervisors? |
| 8. | | To what extent is portfolio use impacted by the fact that training takes place in a workplace setting? |
| Do you have anything to add to what we discussed earlier? | | |
