# Supplementary material for: How does portfolio use affect self-regulated learning in clinical workplace learning: What works, for whom, and in what contexts?
Source: Perspect Med Educ. 2022 Sep 22;11(5):247–57. doi: 10.1007/s40037-022-00727-7 (PMC9582105; doi:10.1007/s40037-022-00727-7)
Supplement: Supplementary file 3 — Electronic supplement 3 Search strings exploratory scoping search [file 40037_2022_727_MOESM3_ESM.docx]

**Electronic supplement 3**Search strings exploratory scoping search

**Executed on July 18^th^, 2018**

Pubmed (35 results)

(("Education"[Mesh] AND (postgrad*[tiab] OR post-grad*[tiab] OR workplace[tiab] OR work-place[tiab] OR work based[tiab] OR vocational[tiab])) OR postgraduate educat*[tiab] OR post-graduate educat*[tiab] OR workplace based educat*[tiab] OR work-place based educat*[tiab] OR work based educat*[tiab] OR vocational educat*[tiab] OR workplace based training[tiab] OR work-place based training[tiab] OR work based training[tiab] OR vocational training[tiab] OR "Education, Medical, Continuing"[Mesh] OR "Education, Medical, Graduate"[Mesh]) AND (portfol*[tiab] OR eportfol*[tiab] OR e-portfol*[tiab] OR port-fol*[tiab] OR eport-fol*[tiab] OR e-port-fol*[tiab])) AND ("Self-Directed Learning as Topic"[Mesh] OR (("Learning"[Mesh] OR learn*[tiab]) AND (self-direct*[tiab] OR selfdirect*[tiab] OR self-regul*[tiab] OR selfregul*[tiab] OR steering[tiab])))

Web of Science (18 results)

TS=((postgraduate OR post-graduate OR “work based” OR “workplace based” OR “vocational education” OR “vocational training”) AND (portfolio OR eportfolio OR e-portfolio) AND (self-directed OR self-regulated OR selfdirected OR selfregulated OR steering))
