# Supplementary material for: How does portfolio use affect self-regulated learning in clinical workplace learning: What works, for whom, and in what contexts?
Source: Perspect Med Educ. 2022 Sep 22;11(5):247–57. doi: 10.1007/s40037-022-00727-7 (PMC9582105; doi:10.1007/s40037-022-00727-7)
Supplement: Supplementary file 4 — Electronic supplement 4 Search strings in-depth literature search [file 40037_2022_727_MOESM4_ESM.docx]

**Electronic supplement 4**Search strings in-depth literature search

**Executed on February 19^th^, 2019**

Pubmed (999 results)

(portfol*[tiab] OR eportfol*[tiab] OR e-portfol*[tiab] OR port-fol*[tiab] OR eport-fol*[tiab] OR e-port-fol*[tiab]) **AND** ("Self-Directed Learning as Topic"[Mesh] OR personal development plan*[tiab] OR self-direct*[tiab] OR selfdirect*[tiab] OR self-regul*[tiab] OR selfregul*[tiab] OR "Formative Feedback"[Mesh] OR "Self-Assessment"[Mesh] OR selfassessment[tiab] OR Lifelong learning[tiab] OR Professional development[tiab] OR Learning cycle*[tiab] OR Learning tool*[tiab] OR educational tool*[tiab] OR Self evaluation*[tiab] OR Self appraisal*[tiab] OR reflect*[tiab] OR (("Learning"[Mesh] OR learn*[tiab]) AND (steering[tiab] OR goals[Mesh] OR goal[tiab] OR goals[tiab] OR feedback[tiab] OR assessment[tiab] OR keeping track[tiab] OR monitor*[tiab] OR progress[tiab])))

CINAHL (786 results)

(MH "Portfolio" OR TI (portfol* OR eportfol* OR e-portfol* OR port-fol* OR eport-fol* OR e-port-fol*) OR AB (portfol* OR eportfol* OR e-portfol* OR port-fol* OR eport-fol* OR e-port-fol*)) **AND** (MH "Self Directed Learning" OR MH "Self Assessment" OR TI ( personal development plan* OR self-direct* OR selfdirect* OR self-regul* OR selfregul* OR Lifelong learning OR Professional development OR Learning cycle* OR Learning tool* OR educational tool* OR Self evaluation* OR Self appraisal* OR reflect* OR self-assessment OR selfassessment ) OR AB ( personal development plan* OR self-direct* OR selfdirect* OR self-regul* OR selfregul* OR Lifelong learning OR Professional development OR Learning cycle* OR Learning tool* OR educational tool* OR Self evaluation* OR Self appraisal* OR reflect* OR self-assessment OR selfassessment ) OR MH “Feedback" OR ((MH "Lifelong Learning" OR MH "Personal Growth" OR MH "Skill Acquisition" OR MH "Skill Retention" OR MH "Learning" OR TI (learn*) OR AB (learn*)) AND (TI ( steering OR goal OR goals OR feedback OR self-assessment OR selfassessment OR keeping track OR monitor* OR progress ) OR AB (steering OR goal OR goals OR feedback OR keeping track OR monitor* OR progress ))))

ERIC (324 results)

(portfolio assessment/ OR ((portfol* or eportfol* or e-portfol* or port-fol* or eport-fol* or e-port-fol*).ti,ab,tw. )) **AND** ((exp medical education/ or "clinical teaching (health professions)"/ or health personnel/ or health sciences/) OR ((medical or clinical or health or healthcare).ti,ab,tw.))

PsycInfo (238 results)

((portfol* or eportfol* or e-portfol* or port-fol* or eport-fol* or e-port-fol*).ti,ab,id.) **AND** (self-evaluation/ OR (personal development plan* or self-direct* or selfdirect* or self-regul* or selfregul*).ti,ab,id. or adult learning/ or Self evaluation/ or selfassessment.ti,ab,id. or Lifelong learning.ti,ab,id. or Professional development.ti,ab,id. or Learning cycle*.ti,ab,id. or Learning tool*.ti,ab,id. or educational tool*.ti,ab,id. or Self evaluation*.ti,ab,id. or Self appraisal*.ti,ab,id. or reflect*.ti,ab,id. or ((exp Learning/ or learn*.ti,ab,id.) and (steering.ti,ab,id. or goal setting/ or goal.ti,ab,id. or goals.ti,ab,id. or feedback.ti,ab,id. or assessment.ti,ab,id. or keeping track.ti,ab,id. or monitor*.ti,ab,id. or progress.ti,ab,id.))) **AND** (exp Medical Education/ OR nursing education/ OR (exp Clinical Methods Training/ or exp Physicians/ or exp Physical Therapists/ or exp Nursing Students/) OR ((medical or clinical or health or healthcare).ti,ab,id.))

Embase (1073 results)

((portfol* or eportfol* or e-portfol* or port-fol* or eport-fol* or e-port-fol*).ti,ab,kw.) AND (self-directed learning/ or personal development plan*.ti,ab,kw. or self-direct*.ti,ab,kw. or selfdirect*.ti,ab,kw. or self-regul*.ti,ab,kw. or selfregul*.ti,ab,kw. or constructive feedback/ or exp Self evaluation/ or selfassessment.ti,ab,kw. or Lifelong learning.ti,ab,kw. or Professional development.ti,ab,kw. or Learning cycle*.ti,ab,kw. or Learning tool*.ti,ab,kw. or educational tool*.ti,ab,kw. or Self evaluation*.ti,ab,kw. or Self appraisal*.ti,ab,kw. or reflect*.ti,ab,kw. or ((exp Learning/ or learn*.ti,ab,kw.) and (steering or goal or goals or feedback or assessment or keeping track or monitor* or progress).ti,ab,kw.))

Web of Science (688 results)

(portfol* or eportfol* or e-portfol* or port-fol* or eport-fol* or e-port-fol*) **AND** ((“personal development plan*” OR self-direct* OR selfdirect* OR self-regul* OR selfregul* OR “formative feedback” OR “Self evaluation*” OR “self assessment*” OR selfassessment OR “Lifelong learning” OR “Professional development” OR “Learning cycle*” OR “Learning tool*” OR “educational tool*” OR “Self appraisal*” OR reflect* OR (learn* AND (steering OR goal OR goals OR feedback OR assessment OR keeping track OR monitor* OR progress)))) **AND** (medical or clinical or health or healthcare)
