# Supplementary material for: How does portfolio use affect self-regulated learning in clinical workplace learning: What works, for whom, and in what contexts?
Source: Perspect Med Educ. 2022 Sep 22;11(5):247–57. doi: 10.1007/s40037-022-00727-7 (PMC9582105; doi:10.1007/s40037-022-00727-7)
Supplement: Supplementary file 5 — Electronic supplement 5 Inclusion criteria in-depth literature search [file 40037_2022_727_MOESM5_ESM.docx]

**Electronic supplement 5**

Inclusion criteria in-depth literature search

- Only primary research papers (quantitative/qualitative/mixed methods) were included.
  - Excluded were:
    - Perspectives
    - Reviews
    - Theoretical pieces
    - Dissertations
    - Books
    - Book reviews
    - Editorials
- The study needed to have a primary focus on (e-)portfolio use.
  - Studies that used (e-)portfolios as data source for other research aims were excluded, e.g. studies that assessed quality of reflection via portfolio entries.
  - Studies that evaluated multiple elements of a curriculum, of which the (e-)portfolio was one component, were excluded.
- The study needed to focus on Self-Regulated Learning/Self-Directed Learning.
  - If the study focused on constructs that are part of SRL/SDL, e.g. reflection, goal setting, it was also included.
- The study needed to concern clinical workplace learning, e.g. rotations or postgraduate trainings.
  - Formal education/training was required; meaning no self-study, (short) courses, etc.
    - Continuing Professional Development was excluded.
  - Combinations between clinical training and classroom education were included.
  - Education/training that focused on topics that are not essential for medical training, e.g. teaching, were excluded.
  - Simulation learning was excluded.
- The study needed to be conducted within the field of medical education.
- The study needed to focus on the effects for the learner (students/trainees).
  - Faculty development was excluded.
- Papers in languages other than English, Dutch, French or German were excluded.
- Papers used for the program theory were excluded.
