# Supplementary material for: How does portfolio use affect self-regulated learning in clinical workplace learning: What works, for whom, and in what contexts?
Source: Perspect Med Educ. 2022 Sep 22;11(5):247–57. doi: 10.1007/s40037-022-00727-7 (PMC9582105; doi:10.1007/s40037-022-00727-7)
Supplement: Supplementary file 6 — Electronic supplement 6 Flowchart of the in-depth literature search and screening proces [file 40037_2022_727_MOESM6_ESM.docx]

**Electronic supplement 6**

Flowchart of the in-depth literature search and screening process

1950 references excluded

78 full-text papers excluded

16 papers included

94 full-text papers assessed

2044 references after the removal of duplicates

4108 references identified through database searching

**ERIC**
324 records

**Web of Science**

688 records

**Embase**
1073 records

**PsycInfo**
238 records

**Pubmed**
999 records

**CINAHL**
786 records
