# Supplementary material for: How does portfolio use affect self-regulated learning in clinical workplace learning: What works, for whom, and in what contexts?
Source: Perspect Med Educ. 2022 Sep 22;11(5):247–57. doi: 10.1007/s40037-022-00727-7 (PMC9582105; doi:10.1007/s40037-022-00727-7)
Supplement: Supplementary file 7 — Electronic supplement 7 Characteristics and qualitative rigor evaluation of the 16 included papers [file 40037_2022_727_MOESM7_ESM.docx]

**Electronic supplement 7**

Characteristics and qualitative rigor evaluation of the 16 included papers

| Author, year [ref] | Country | Educational setting | Study aim | Study design, methods | Rigor evaluation^a,b^ |
| --- | --- | --- | --- | --- | --- |
| Amsellem-Ouazana et al., 2006 [31] | France | Undergraduate education: clinical years. | Evaluation of the portfolio used. | Quantitative design,  Questionnaire. | 1 2 3 ○○○ |
| Berger et al, 2011 [32] | Canada | Postgraduate education: pediatric specialty training. | Evaluation of the portfolio used. | Qualitative design, Questionnaire. | 1 2 3 ○○○ |
| Brown et al, 2013 [33] | England | Postgraduate education: specialty training. | Exploration of the factors that trigger refection and why documented evidence does not always encapsulate practice. | Qualitative design, Semi-structured interviews. | 1 2 3 ●●● |
| Elango et al, 2005 [34] | Malaysia | Undergraduate education: clinical years. | Gaining insight into the perspectives of students concerning portfolio learning. | Mixed methods design,  Feedback was used to compose a questionnaire. | 1 2 3 ○○○ |
| Fida et al, 2018 [35] | Saudi Arabia | Undergraduate education: non-clinical and clinical years. | Evaluation of students’ perceptions of the effectiveness of portfolios in developing reflective practice. | Mixed methods design,  Content analysis and questionnaire. | 1 2 3 ○○○ |
| Fu et al, 2019 [36] | Taiwan | Postgraduate education: postgraduate year 1 (training between masters and specialty training). | Understanding trainees’ Feedback Seeking Behaviors in the context of an e-portfolio. | Qualitative design,  Semi-structured interviews. | 1 2 3 ●●● |
| Haffling et al, 2010 [37] | Sweden | Undergraduate education: clinical year. | Investigation of the presence of professional competence in students’ reflections and an evaluation of students’ satisfaction with the portfolio. | Mixed methods design,  Content analysis and questionnaire. | 1 2 3 ○○● |
| Halder et al, 2012 [38] | England | Postgraduate education: psychiatry specialty training. | Evaluation of the portfolio used. | Mixed methods design,  Questionnaire. | 1 2 3 ○○○ |
| Hrisos et al, 2008 [39] | England | Postgraduate education: foundation program (training that all newly graduated doctors follow). | Exploration of the experiences of trainees and supervisors concerning the learning portfolio. | Mixed methods design, Interviews were used to compose a questionnaire. | 1 2 3 ○○● |
| Jenkins et al, 2013 [40] | South-Africa | Postgraduate education: general practitioner specialty training. | Exploration of the views of registrars and supervisors regarding the portfolio’s educational impact, acceptability, and perceived usefulness for assessment of competence. | Qualitative design,  Semi-structured interviews. | 1 2 3 ●●● |
| Jenkins et al, 2013 [41] | South-Africa | Postgraduate education: general practitioner specialty training. | Establishing engagement with the portfolio and in particular whether  users found the portfolio acceptable and practical to  complete, useful for learning, and useful for summative  assessment purposes. | Mixed methods design,  Questionnaire. | 1 2 3 ○○○ |
| Kjaer et al, 2006 [42] | Denmark | Postgraduate education: general practitioner specialty training. | Exploration of how trainees perceived the use of an online  portfolio and how it affected their learning. | Mixed methods design,  Questionnaire. | 1 2 3 ●●○ |
| Sheng et al, 2018 [43] | United States | Undergraduate education: 3rd and 4th year students rotating at the emergency department. | Evaluation of the portfolio used. | Qualitative design,  Semi-structured interviews. | 1 2 3 ●●● |
| Snadden et al, 1996 [44] | Scotland | Postgraduate education: general practitioner specialty training. | Finding out to what extent portfolio learning was useful  and acceptable to trainers and GP registrars | Qualitative design, Action research. | 1 2 3 ○●○ |
| Snadden et al, 1998 [45] | Scotland | Postgraduate education: general practitioner specialty training. | Introducing and monitoring of a reflective learning strategy, mapping the usefulness of a portfolio model in terms of  general practice vocational training, and evaluating the effectiveness of an external facilitator. | Qualitative design, Participatory action research. | 1 2 3 ●●○ |
| Webb et al, 2012 [46] | United States | Postgraduate education: surgery specialty training | Determining the effectiveness of  the SLIP program in its current configuration promoting self-reflection  and practice-based learning. | Mixed methods design,  Questionnaire, focus group, log data and content analysis. | 1 2 3 ●●○ |

a The three criteria for our rigor evaluation were (1) A clear statement of, and rationale for, the research question/aims, (2) Design and study methods are appropriate to answer the research question, (3) Study findings and conclusions are supported by the data.
b In case the paper fulfilled a rigor criterium this is indicated with a black circle (●). In case the paper did not fulfil the rigor criterium this indicated with an open circle (○).
